# Protection of Bovine Mammary Epithelial Cells from Hydrogen Peroxide-Induced Oxidative Cell Damage by Resveratrol

**DOI:** 10.1155/2016/2572175

**Published:** 2015-12-28

**Authors:** Xiaolu Jin, Kai Wang, Hongyun Liu, Fuliang Hu, Fengqi Zhao, Jianxin Liu

**Affiliations:** ^1^Institute of Dairy Science, Key Laboratory of Molecular Animal Nutrition, Ministry of Education, Zhejiang University, Hangzhou 310058, China; ^2^College of Animal Sciences, Zhejiang University, Hangzhou 310058, China; ^3^Laboratory of Lactation and Metabolic Physiology, Department of Animal Science, University of Vermont, Burlington, VT 05405, USA

## Abstract

The mammary epithelial cells (MECs) of high-producing dairy cows are likely to be subject to oxidative stress (OS) due to the intensive cell metabolism. The objectives of this study were to investigate the cytoprotective effects of resveratrol against hydrogen peroxide- (H_2_O_2_-) induced OS in cultured bovine MECs (MAC-T). Pretreatment of MAC-T cells with resveratrol could rescue the decrease in cell viability and resulted in lower intracellular reactive oxygen species (ROS) accumulation after H_2_O_2_ exposure. Resveratrol helped MAC-T cells to prevent H_2_O_2_-induced endoplasmic reticulum stress and mitochondria-related cell apoptosis. Moreover, resveratrol induced mRNA expression of multiple antioxidant defense genes in MAC-T cells under normal/oxidative conditions. Nuclear factor erythroid 2-related factor 2 (Nrf2) was required for the cytoprotective effects on MAC-T cells by resveratrol, as knockdown of Nrf2 significantly abolished resveratrol-induced cytoprotective effects against OS. In addition, by using selective inhibitors, we further confirmed that the induction of Nrf2 by resveratrol was mediated through the prolonged activation of PI3K/Akt and ERK/MAPK pathways but negatively regulated by p38/MAPK pathway. Overall, resveratrol has beneficial effects on bovine MECs redox balance and may be potentially used as a therapeutic medicine against oxidative insult in lactating animals.

## 1. Introduction

Oxidative stress has been implicated in the human disease development [1]. It occurs when reactive oxygen species (ROS) production exceeds the antioxidant capacity of cells, thus leading to induction of lipid peroxidation and protein modification and subsequently cellular dysfunction and diseases [2]. Antioxidant compounds derived from food components can protect cells against oxidative stress. These healthy benefits are attributed to direct scavenging free radicals or indirect increasing endogenous cellular antioxidant potential, such as through the activation of nuclear factor erythroid 2-related factor 2 (Nrf2).

Nrf2 is a master cellular sensor for ROS and its activation regulates gene expression of cellular defense enzymes and certain antioxidant proteins through the antioxidant response element (ARE) [3]. In physiological state, Nrf2 is retained within the cytosol by its inhibitory partner, a cysteine-rich anchor protein called Kelch-like ECH-associated protein 1 (Keap1). The binding of Nrf2 to Keap1 forms an E3 ubiquitin ligase-based complex and leads to their rapid degradation by the ubiquitin-proteasome system. Previous works revealed that ROS result in the accumulation of Nrf2 and facilitate its nuclear translocation, initiating the transcription of ARE-contained genes that are involved in several key events against oxidative stress, such as cysteine uptake transporter (xCT), NADPH-Quinone oxidoreductase 1 (NQO1), and hemeoxygenase 1 (HO-1). Several exogenous/endogenous chemicals, including NO, nitrofatty acids, and 4-hydroxynonenal, are known to induce ARE-containing genes through Nrf2 activation [4].

Oxidative stress may be involved in several pathological conditions in farm animals, such as thermal and physical discomfort, injuries [5], colitis [6], and sepsis [7] in pigs, pneumonic pasteurellosis in sheep [8], pneumonia in foals [9], and demodicosis in dogs [10]. High-producing dairy cows are likely subject to altered redox balance due to high metabolic rates and physiological adaptations [11]. The mammary epithelial cells (MECs) of lactating cows undergo intensive cell metabolism and accumulate a large amount of free radicals, like ROS. Previous studies in mouse mammary gland found that the ductal cells contain higher level of ROS than the myoepithelial cells [12], and ROS produced by MECs could have long-term consequences during the lactation, initiating luminal but not basal cell death in cultured human mammary alveolar structures [13]. In addition, Schogor et al. found a linear increase in Nrf2 mRNA abundance in mammary tissue of cows with flax meal supplementation [14], which suggested that Nrf2 might participate in the promotion of cellular antioxidant potentials of MECs. Although studies have been carried out to supplement animals with certain exogenous antioxidants for protecting dairy cows against oxidative stress [15, 16], it is still unknown whether or how these antioxidants have direct protective effects on MECs.

Resveratrol (trans-3,5,4′-trihydroxystilbene) is a natural polyphenolic compound that is present in many plant species, including grapevines and berries [17, 18]. It has been shown to have an efficient antioxidant property by both* in vitro* [19, 20] and* in vivo* studies [21, 22], but its antioxidant role has not been well understood in MECs of dairy cattle. The purposes of this study were to (i) investigate the oxidative damaging effects of hydrogen peroxide (H_2_O_2_) on growth of bovine MECs* in vitro*, (ii) determine whether resveratrol has a cytoprotective effect on MECs by restoring the cell redox state during oxidative stress, and (iii) study the underlying mechanisms of resveratrol's possible antioxidative effects.

## 2. Materials and Methods

### 2.1. Chemicals and Regents

Chemicals, including resveratrol, tBHQ, and 2′,7′-dichlorofluorescin diacetate (DCFH-DA), and alkaline phosphatase- (AP-) conjugated secondary antibody (anti-rabbit IgG) were purchased from Sigma-Aldrich (St. Louis, MO, USA). Primary antibodies against phosphor-Akt (pS473), phosphor-ERK1 (pY204)/ERK2 (pY187), phosphor-JNK1 (pT183)/JNK2 (pT183)/JNK3 (pT221), and *β*-tubulin were purchased from Epitomics (Burlingame, CA, USA). Antibody against phospho-p38 (Thr 180/Tyr 182) was purchased from Cell Signaling Technology (Danvers, MA, USA), and anti-Nrf2 antibody was purchased from Abcam (Cambridge, Massachusetts, USA). Specific inhibitors, PD98059 (for ERK1/2 signaling), LY294002 (for Akt signaling), SP600125 (for JNK signaling), and SB203580 (for p38 signaling), were obtained from Selleckchem (Houston, TX, USA). Other analytical grade chemicals were purchased from Sangon Biotechnology Co. (Shanghai, China).

### 2.2. Cell Culture and Treatment

Bovine MEC line MAC-T cells [23] were maintained in high-glucose Dulbecco's modified Eagle's medium (HG-DMEM, Pierce Hyclone, Fremont, CA, USA) supplemented with 100 U/mL of penicillin, 100 *μ*g/mL streptomycin, and 10% (V/V) heat-inactivated fetal bovine serum (FBS, Gibco, Carlsbad, CA, USA) at 37°C and 5% CO_2_ in a humidified incubator. To establish* in vitro* oxidative stress model, H_2_O_2_ was applied to MAC-T cells. We firstly diluted 30% H_2_O_2_ to 1 M stock using sterilized PBS (100 *μ*L 30% H_2_O_2_ was diluted with 870.3 *μ*L PBS). For the cell treatment, indicated concentrations of resveratrol were applied to the cells for 2 h pretreatment. Meanwhile, 1 M H_2_O_2_ was further diluted with cell culture medium at required concentrations and then added into culture plates and incubated for required time periods. All of required H_2_O_2_ solutions were made freshly before being used.

### 2.3. Cell Viability Assay

Cell viability assay was performed using the CCK-8 kit (Dojindo, Kumamoto, Japan) according to the manufacture's instruction. Briefly, 10 × 10^4^/mL MAC-T cells were seeded into 96-well culture plates. After 24 h incubation, cells in each well were incubated with 10 *μ*L of CCK-8 at 37°C for 2 h before measuring the OD at 450 nm with a microplate reader (M5, MD, USA). Cell viability was also confirmed by trypan blue exclusion.

### 2.4. Detection of Intracellular ROS

ROS production was determined by carboxy-H2DCF-DA staining assay [24]. Briefly, after treatment, MAC-T cells were incubated with 10 *μ*M carboxy-H2DCF-DA at 37°C for 30 min. Cells (1 × 10^6^) were then resuspended in phosphate-buffered saline (PBS) and analyzed for fluorescence using flow cytometry. The percentage of fluorescence-positive cells was recorded on a FACSCalibur flow cytometer (BD Biosciences, San Diego, CA, USA) using excitation and emission filters of 488 and 530 nm, respectively.

### 2.5. Immunofluorescence Staining

For immunofluorescence staining, MAC-T cells were seeded in laser confocal petri dishes (Coring Life Science, Lowell, CA, USA). After the treatment, the cells were washed 3 times with PBS, fixed with ice-cold methanol and acetone (v : v = 1 : 1) solution for 30 min, and permeabilized with 0.5% PBS-Triton for 30 min. Then, the cells were sequentially incubated in PBS containing 10% normal goat serum to block nonspecific protein-protein interactions, in primary rabbit anti-Nrf2 antibody (1 : 200 dilution) overnight at 4°C, and in the secondary FITC-conjugated goat anti-rabbit IgG (1 : 500 dilution, Mutisciences, Hangzhou, China) for 1 h at 37°C in the dark, respectively. After 3 rinses in PBS, the nucleuses of the cells were stained with DAPI for 5 minutes. Finally, cells were visualized with a confocal laser microscopy (Leica, TCS SP5, Germany) and images were taken under an Olympus FLUOVIEW FV1000 microscope.

### 2.6. RNA Isolation and Quantitative Real-Time PCR (qPCR)

Total RNA was extracted with the RNA pure Kit (Aidlab Biotechnologies Co., Ltd., Beijing, China) according to the manufacturer's procedures. Reverse transcription of 1 *μ*g total RNA was performed using the PrimeScript RT reagent kit (TaKaRa, Dalian, China). The reverse transcription product was diluted 1 : 10 and used as cDNA template for qPCR analysis. qPCR was carried out in a 7500c real-time PCR detection system (Applied Biosystems, Carlsbad, CA, USA) with the SYBR premix EX Taq (TaKaRa) following the manufacturer's instructions using a standard two-step reaction [25]. Expression of the housekeeping gene *β*-actin was used for normalization of other genes' expression. qPCR primers were designed to flank introns with the Primer 5 software (Premier Biosoft International, Palo Alto, CA) and listed in Supplemental Table 1 in Supplementary Material available online at http://dx.doi.org/10.1155/2016/2572175.

### 2.7. Preparation of Cell Lysates and Western Blot Analysis

To obtain total protein lysates, the MAC-T cells were lysed on ice for 10 min using a cell lysis buffer containing 50 mM Tris-Cl (pH 7.5), 150 mM NaCl, 0.5% NP-40, 10% glycerol, 2 mM  DTT, 1 mM  leupeptin, and 1 mM PMSF. All lysates were collected by scraping the culture dishes with cell scrapers and centrifuged at 4°C at a speed of 12.000 g for 10 min to remove cell debris. Protein concentrations were measured by BCA protein assay kit (Beyotime, Nanjing, China). Equal amounts of cellular proteins (20 *μ*g) were mixed with Laemmli's sample buffer and boiled at 95°C for 5 min [26]. The proteins were separated by 12–15% SDS-PAGE and transferred to polyvinylidene fluoride (PVDF) membranes (Millipore, USA). The membranes were incubated in 5% skim milk in Tris-buffered saline containing Tween 20 (TBST, 20 mM Tris-Cl, pH 7.4, 150 mM NaCl, and 0.02% Tween 20) for 30 min at room temperature to block the nonspecific binding sites. Then, the blots were incubated with diluted primary antibodies (1 : 1.000 dilution for phosphor-Akt, phospho-p38, and phosphor-JNK1/JNK2/JNK3, 1 : 2.000 dilution for phosphor-ERK1/ERK2, and 1 : 5.000 dilution for *β*-tubulin) for 1 h at room temperature. After washing with TBST, the blots were incubated with an alkaline phosphatase-conjugated secondary antibody (1: 10.000 dilutions in TBST) for 1 h at room temperature. After three times of washing with TBST, the immunoreactive protein bands on the membranes were developed for 3 min in 10 mL color development buffer (100 mM Tris-Cl, pH 9.5, 50 mM NaCl, and 5 mM MgCl_2_) mixed with 100 *μ*L NBT/BCIP solution (18.75 mg/mL nitro blue tetrazolium chloride (NBT) and 9.4 mg/mL 5-bromo-4-chloro-3-indolyl phosphate toluidine salt (BCIP in 67% DMSO, v/v)). The Western blotting results were quantified using Quantity One software [27].

### 2.8. Transient Transfection and Nrf2 Small RNA Interference

Three candidate siRNAs targeting the coding region of Nrf2 mRNA and one negative control siRNA (Supplemental Table 2) were synthesized by Biomics Biotechnologies Co. (Nanjing, China). The effectiveness and specificity of the siRNAs were examined in MAC-T cells by quantitative analysis of expression of Nrf2 at 24 h after siRNA transfection. siNrf2-3 was shown to be mostly effective and thus used in subsequent experiments (81% knockdown, Supplemental Figure 1). Transfection was performed with RNAiMAX Lipofectamine 2000 reagent (Invitrogen, Carlsbad, CA, USA) according to the manufacturer's instruction. Briefly, each siRNA construct was diluted in Opti-MEM Reduced Serum Medium (Invitrogen) and then mixed with an equal volume of Lipofectamine. After incubation for 15 min, the siRNA/Lipofectamine solution was added directly to cells without removing the original cell media. The final siRNA concentration was 50 nM. After 24 h, the siRNA was removed from the cells, and cells were used for subsequent analysis or treatment.

### 2.9. Statistical Analysis

Data are expressed as the means ± SD for the indicated number of independently performed experiments. One-way analysis of variance (ANOVA) followed by Student-Newman-Keuls (SNK) multiple-comparison test was used to determine statistical significance for multiple comparisons, and Student's* t*-test was used for comparisons of two groups. ^*∗*^ *p* < 0.05 was accepted as statistically significant. All statistical tests were carried out using SPSS 17.0.

## 3. Results

### 3.1. Effects of Resveratrol on H_2_O_2_-Induced MAC-T Cell Death

**Figure 1:**
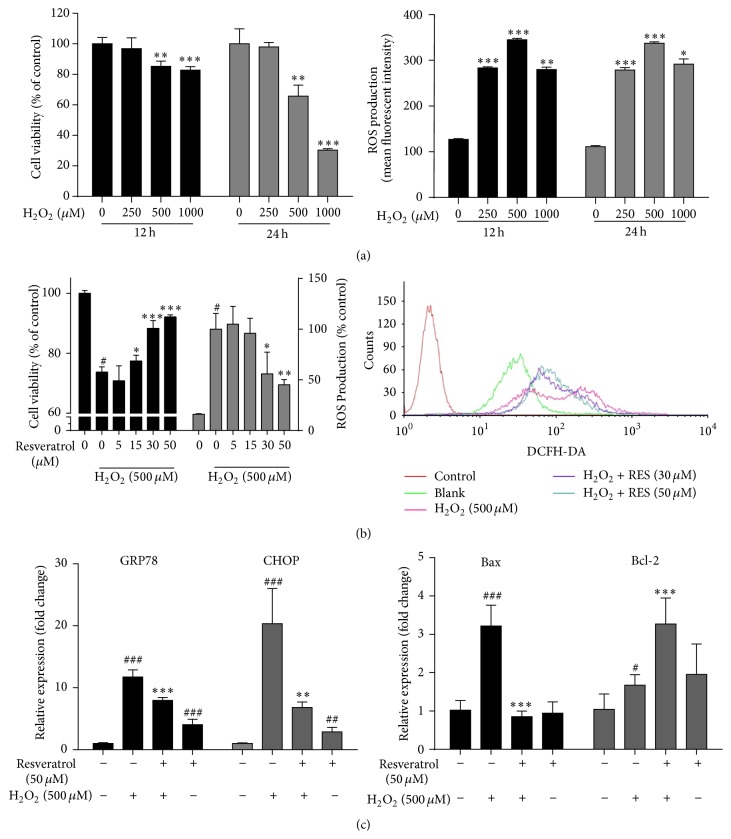
Protective effects of resveratrol against H_2_O_2_-induced MAC-T cell death and ROS production. (a) MAC-T cells were treated with increasing concentrations of H_2_O_2_ (0, 250, 500, and 1000 *µ*M) for 12 h or 24 h. Cell viability was measured by CCK-8 assay (left panel), and ROS concentration was measured by flow cytometer after being loaded with 10 *µ*M DCFH-DA fluorescence probe for 30 min (right panel). ^*∗*^ *p* < 0.05, ^*∗∗*^ *p* < 0.01, and ^*∗∗∗*^ *p* < 0.001 significantly different from untreated cells. (b) MAC-T cells were pretreated with the indicated concentrations of resveratrol for 2 h, followed by H_2_O_2_ (500 *μ*M) challenge for 24 h. Then the cell viability (black bars) and ROS concentration (grey bars) were determined (left panel). A representative flow cytometric histogram is shown (right panel). (c) MAC-T cells were pretreated with or without 50 *μ*M of resveratrol for 2 h and then treated with or without 500 *μ*M of H_2_O_2_. The mRNA expression of the endoplasmic reticulum stress markers GRP 78 and CHOP (4 hours after H_2_O_2_ treatment) and mitochondria-related cell apoptosis markers Bax and Bcl-2 (24 h after H_2_O_2_ treatment) was analyzed by quantitative real-time PCR. Data are represented as mean ± SD from three independent experiments. # means significantly different from untreated cells. *∗* means significantly different from H_2_O_2_-treated cells.

Treatment of MAC-T cells with increasing concentrations of H_2_O_2_ (0–1000 *µ*M) for 12 h or 24 h showed time- and dose-dependent inhibition of cell survival (Figure 1(a), left panel), accompanied with sharp increases of ROS (Figure 1(a), right panel). The treatment of cells with 500 *µ*M of H_2_O_2_ for 24 h, a condition that decreased the cell viability to 66% and increased ROS 2.7-fold, was chosen in subsequent experiments of assessing the antioxidative effects of resveratrol in MAC-T cells. Pretreatment of MAC-T cells with 0–50 *µ*M of resveratrol, at which resveratrol itself showed no effect on MAC-T cell growth (Supplemental Figure 2), significantly inhibited H_2_O_2_-induced cell death and ROS increase at a dose-dependent manner (Figure 1(b)). Compared to H_2_O_2_-treated MAC-T cell, 50 *µ*M of resveratrol pretreatment significantly decreased 55% of ROS production (*p* < 0.01).

### 3.2. Resveratrol Rescued H_2_O_2_-Elicited Endoplasmic Reticulum (ER) Stress and Mitochondria-Related Cell Apoptosis in MAC-T Cells

In MAC-T cells, H_2_O_2_ strongly enhanced endoplasmic reticulum (ER) stress markers GRP78 and CHOP mRNA expression in a time-dependent manner, and the induction was peaked at 4 h of the treatment, indicating the effects of H_2_O_2_ on ER stress (Supplemental Figure 3). However, after the pretreatment of the cells with 50 *µ*M resveratrol, the H_2_O_2_-induced expression of GRP78 and CHOP was significantly suppressed (Figure 1(c), left panel). Similarly, resveratrol also exerted potent antiapoptotic effects by downregulating Bax expression and upregulating Bcl-2 expression in MAC-T cells undergoing oxidative stress (Figure 1(c), right panel).

### 3.3. Resveratrol Induces Expression of Multiple Antioxidant Genes under the Normal or Oxidative Conditions in MAC-T Cells

**Figure 2:**
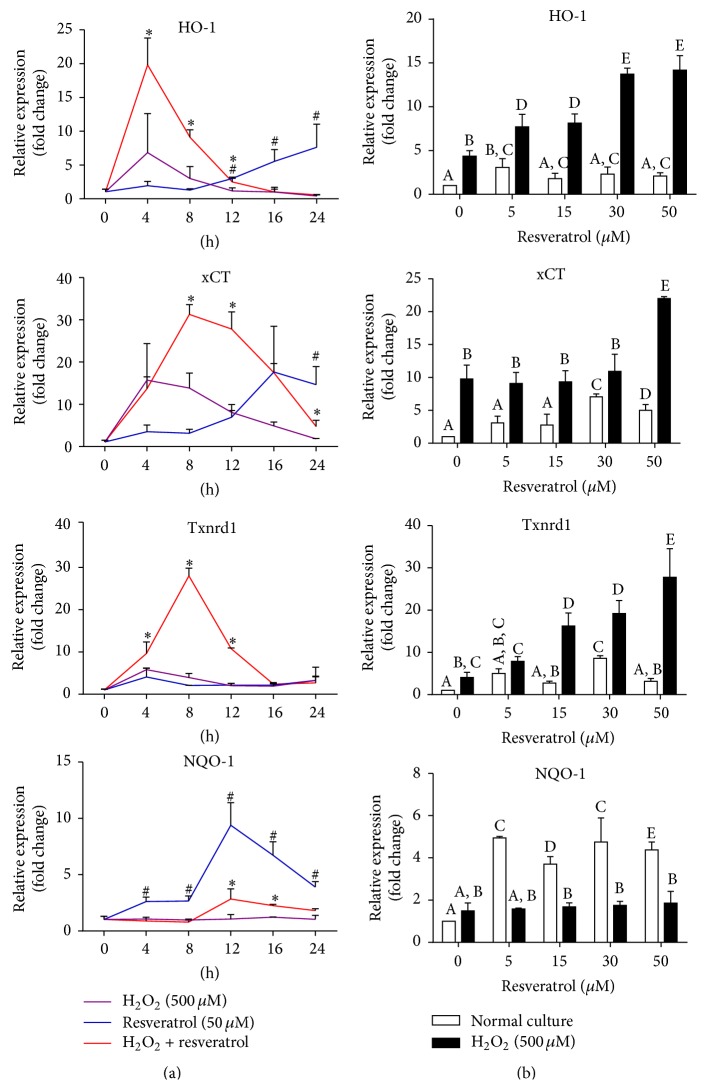
Resveratrol induced gene expressions of multiple antioxidant genes under normal and oxidative conditions. (a) MAC-T cells were treated with resveratrol (50 *µ*M) alone (normal condition) or pretreated with or without resveratrol (50 *µ*M) for 2 h and then treated with 500 *μ*M H_2_O_2_ (oxidative condition) for the indicated time periods. mRNA expression of HO-1, xCT, Txnrd, and NQO-1 genes was analyzed by real-time PCR. Data are represented as mean ± SD from three experiments. Individual treatments were compared using two-tailed Student's* t-test*: ^#^ *p* < 0.05 (resveratrol group was compared with H_2_O_2_ control group at specified time points); ^*∗*^ *p* < 0.05 (resveratrol + H_2_O_2_ group was compared with H_2_O_2_ control group at specified time points); (b) MAC-T cells were pretreated with different doses (0–50 *µ*M) of resveratrol for 2 h and then treated with or without H_2_O_2_ (500 *μ*M) for 8 h. Gene expression was measured by real-time PCR. Data are shown as mean ± SD from three independent experiments. Data were analyzed by one-way ANOVA with the Student-Newman-Keuls method. The means with different superscripts are significantly different (*p* < 0.05).

Because resveratrol has been shown to have antioxidant effect by inducing several antioxidant defense enzymes in several cell lines [28–30], we studied whether it also does so in MAC-T cells. We analyzed the mRNA expression of various genes involved in the detoxification of ROS in MAC-T cells treated with different doses (0–50 *µ*M) of resveratrol for various times (0–24 h), under the normal or oxidative conditions (Figure 2). Under oxidative conditions (with 500 *μ*M H_2_O_2_ treatment), pretreatment of cells with resveratrol (50 *µ*M) significantly increased mRNA expressions of HO-1 (peaked at 4 h), xCT, and thioredoxin reductase (Txnrd) (both peaked at 8 h), whereas, in normal condition (without H_2_O_2_ treatment), resveratrol gradually upregulated mRNA expression of HO-1 (peaked at 24 h), xCT (peaked at 16 h), and NQO-1 (peaked at 12 h) at much later times. We next selected 8 h H_2_O_2_ exposure time to further investigate the dose effects of resveratrol on the mRNA expression of these genes. As shown in Figure 2(b), HO-1, xCT, and Txnrd mRNA expressions were strongly induced by resveratrol in a dose-dependent manner under oxidative condition. Pretreatment of resveratrol has no significant effect on NQO-1 mRNA expression in H_2_O_2_ treated cells but has strong inductive effects in normal condition. Expressions of some other genes of antioxidant defense enzymes, including superoxide dismutase (SOD), glutamate cysteine ligase catalytic subunit (GCLC), and glutathione reductase (GSR), were not affected by resveratrol treatment (data not shown).

### 3.4. The Cytoprotective Effects of Resveratrol against Oxidative Stress Were Dependent on the Induction of Nrf2 in MAC-T Cells

**Figure 3:**
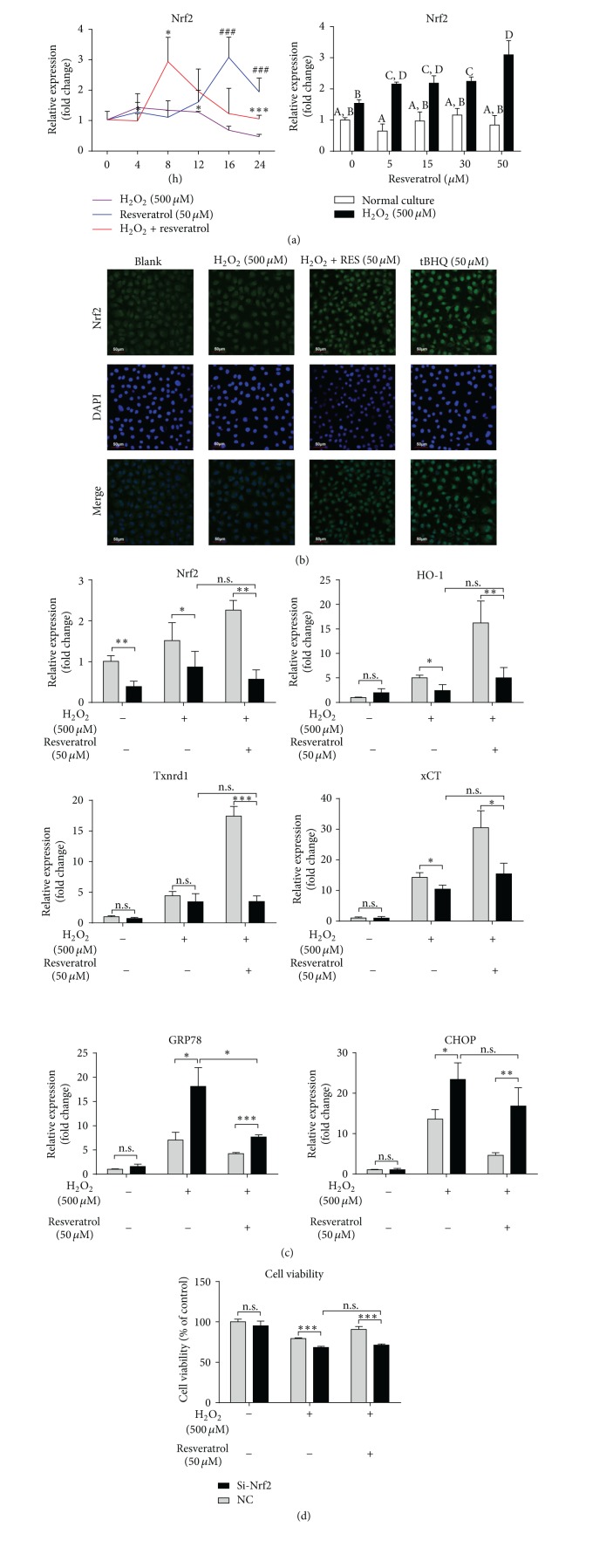
The cytoprotective effects of resveratrol against oxidative stress were dependent on the induction of Nrf2 in MAC-T cells. (a) Left panel: quantitative real-time PCR analysis of Nrf2 mRNA in MAC-T cells pretreated with or without resveratrol for 2 h and then treated with or without H_2_O_2_ for 0–24 h. ^#^ *p* < 0.05 (resveratrol group was compared with H_2_O_2_ control group at specified time points); ^*∗*^ *p* < 0.05 (resveratrol + H_2_O_2_ group was compared with H_2_O_2_ control group at specified time points); Right panel: mRNA expression of Nrf2 in MAC-T cells pretreated with 0–50 *µ*M of resveratrol for 2 h and then treated with (H_2_O_2_ group) or without (normal condition group) 500 *µ*M of H_2_O_2_ for 8 h. The means with different superscripts are significantly different (*p* < 0.05). (b) Immunofluorescence staining of Nrf2 in MAC-T cells treated with or without resveratrol (RES) for 2 h or H_2_O_2_ for 8 h. DAPI staining was performed to stain the nucleus. Treatment of cells with tBHQ (50 *µ*M) for 8 h served as a positive control for Nrf2 translocation. (c) mRNA expression of Nrf2, HO-1, TrxR-1, xCT, GRP78, and CHOP in MAC-T cells transfected with either a Nrf2 siRNA (Si-Nrf2-3) or a control siRNA (NC) for 12 h and with or without resveratrol pretreatment for 2 h followed by H_2_O_2_ treatment for additional 8 h. (d) Cell viability assay in MAC-T cells transfected with either a Nrf2 siRNA (Si-Nrf2-3) or a control siRNA (NC) and with or without resveratrol pretreatment for 2 h and subsequent H_2_O_2_ treatment for additional 12 h. Reported values are the means ± SD from three experiments, ^*∗*^ *p* < 0.05, ^*∗∗*^ *p* < 0.01, and ^*∗∗∗*^ *p* < 0.001.

We next examined whether resveratrol was able to induce Nrf2 expression in MAC-T cells. As shown in Figure 3(a), resveratrol strongly upregulated Nrf2 mRNA expression in a time- (left panel) and dose- (right panel) dependent manner in oxidative condition. Resveratrol could also induce Nrf2 expression in normal condition, but the induction was much slower than in the oxidative condition (comparing the resveratrol group with the H_2_O_2_ plus resveratrol group in Figure 3(a), left panel). In addition, immunofluorescence staining showed that resveratrol stimulated Nrf2 nuclear translocation in MAC-T cells, similar to tBHQ, a well-known Nrf2 activator (Figure 3(b)).

To further elucidate the role of Nrf2 in the cytoprotective effects of resveratrol against oxidative stress, we transfected MAC-T cells with an Nrf2 siRNA. Twelve hours after the transfection, significant decreases in Nrf2 mRNA levels were observed in MAC-T cells treated with or without H_2_O_2_ and resveratrol in comparison to the cells transfected with a control siRNA (Figure 3(c)). Upon H_2_O_2_ exposure, the induction of HO-1, Txnrd, and xCT mRNA by resveratrol treatment was significantly decreased to 72%, 20%, and 50%, respectively, in the cells transfected with Nrf2 siRNA compared to a control siRNA (Figure 3(c)). Interestingly, we also found that Nrf2 knockdown led to a rapid increase of GPR78 and CHOP expression in the cells challenged with H_2_O_2_; however, resveratrol still showed an inhibitory effect on the GPR78 expression in Nrf2 siRNA-transfected cells (Figure 3(c)). Furthermore, knockdown of Nrf2 abolished the protective effect of resveratrol against H_2_O_2_-induced cell viability decrease (Figure 3(d)).

### 3.5. Effects of H_2_O_2_ Exposure and Resveratrol Treatment on MAPK and Akt Signaling Pathways in MAC-T Cells

**Figure 4:**
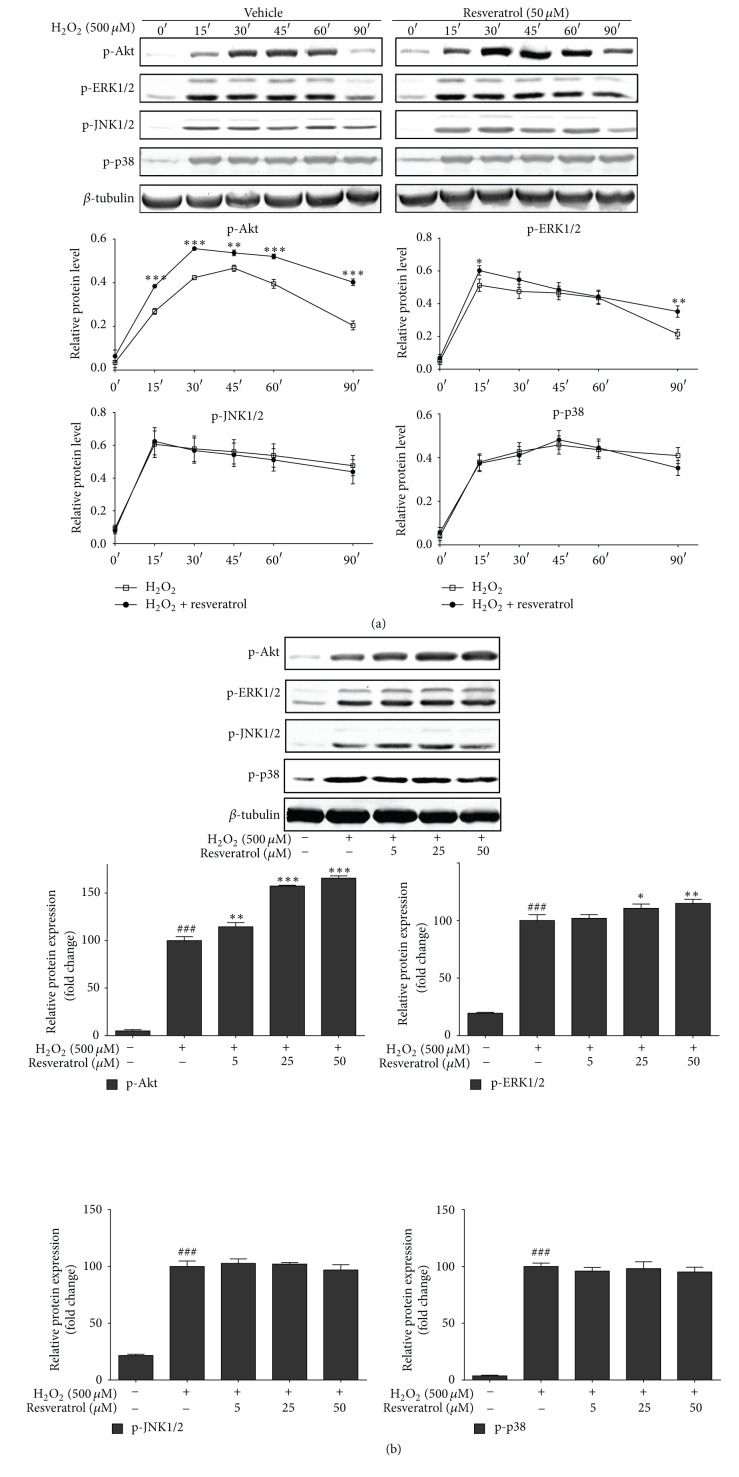
Resveratrol pretreatment prolonged the phosphorylation of Akt and ERK, but not JNK and p38, in H_2_O_2_-treated MAC-T cells. (a) MAC-T cells were preincubated with or without resveratrol (50 *μ*M) for 2 h and then treated with H_2_O_2_ (500 *μ*M) for the indicated time points (0, 15, 30, 45, 60, and 90 min). (b) MAC-T cells were pretreated with vehicle or various concentrations of resveratrol for 2 h and then treated with H_2_O_2_ (500 *μ*M) for 90 min. Phosphorylated Akt, ERK1/2, JNK1/2, and p38 were analyzed by Western blot analysis and quantified. Representative Western blots are shown. *β*-tubulin was used as a loading control. Quantified data are expressed in arbitrary units as mean ± SD of three experiments. # means significantly different from untreated cells. *∗* means significantly different from H_2_O_2_-treated cells.

The effects of resveratrol on activating MAPK, Akt, JNK, and p-38 signaling pathways in MAC-T cells under oxidative conditions (500 *μ*M H_2_O_2_ treatment) were investigated by examining the changes of phosphorylated JNK1/2, ERK, p38, and Akt. As shown in Figure 4(a), JNK1/2, ERK1/2, p38, and Akt were all rapidly activated after treatment with H_2_O_2_, starting from 15 min. More importantly, pretreatment with 50 *μ*M resveratrol resulted in an appreciable prolonged upregulation in phosphorylated ERK and Akt but had no effects on the phosphorylation of p38 and JNK.

### 3.6. Activation of Akt and ERK, but Not p38, Is Required for Resveratrol-Mediated Cytoprotective Effects in MAC-T Cells

**Figure 5:**
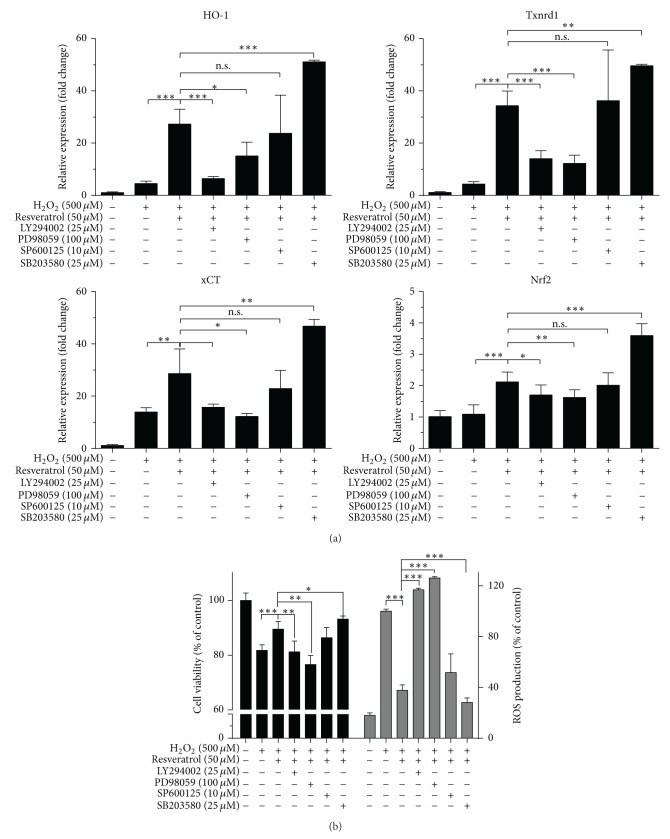
ERK and Akt pathway activations mediate cytoprotective effects of resveratrol against oxidative stress. MAC-T cells were preincubated with or without specific kinase inhibitors LY294002 (25 *μ*M), PD98059 (100 *μ*M), SP600125 (10 *μ*M), or SB203580 (25 *μ*M) for 1 h and then treated with or without resveratrol for another 2 h, followed by H_2_O_2_ exposure. mRNA expression of HO-1, TrxR-1, xCT, and Nrf2 was measured 8 h after H_2_O_2_ stimulation (a) and cell viability and ROS levels were analyzed 12 h after H_2_O_2_ stimulation (b). Values are the means ± SD from three experiments, ^*∗*^ *p* < 0.05, ^*∗∗*^ *p* < 0.01, and ^*∗∗∗*^ *p* < 0.001.

To further study the signaling pathways of resveratrol function, we used selective inhibitors for various signaling pathways in MAC-T cell cultures: LY294002 (for Akt signaling), PD98059 (for ERK1/2 signaling), SP600125 (for JNK signaling), and SB203580 (for p38 signaling). As shown in Figure 5(a), resveratrol-induced Nrf2, HO-1, Txnrd, and xCT gene expression were potently inhibited by PD98059 and LY294002. In addition, these two inhibitors significantly abrogated the ROS scavenging effects by resveratrol (Figure 5(b), right). Furthermore, PD98059 and LY294002 also abolished the cytoprotective effects by resveratrol on H_2_O_2_-challenge (Figure 5(b), left). However, inhibition of p38 signaling by SB203580 even promoted the cytoprotective and ROS scavenging effects by resveratrol. Meanwhile, SB203580 increased the mRNA expression of Nrf2, HO-1, Txnrd-1, and xCT.

## 4. Discussion

**Figure 6:**
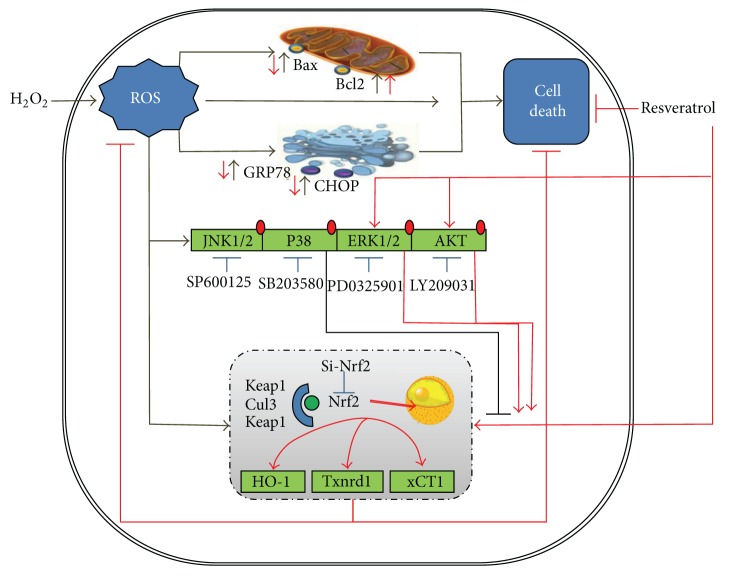
Schematic diagram summarizing the mechanisms underlying the protective effect of resveratrol against H_2_O_2_-induced oxidative stress in MAC-T cells (detailed in Section 4). H_2_O_2_ mediated oxidative stress in MAC-T cells and enhanced the accumulation of ROS. ROS also mediated the upregulations of mitochondria-mediated cell apoptosis genes and ER stress genes, leading to cell death. Resveratrol reduced ROS production by H_2_O_2_ and induced expressions of several antioxidant-stress response genes (HO-1, Txnrd-1, and xCT) at Nrf2 dependent manner. Nrf2-related antioxidant-stress response genes induction by resveratrol during H_2_O_2_ mediated oxidative stress involved stress response protein kinases (mainly PI3K/Akt and ERK/MAPK) but was negatively regulated by p38 MAPK.

In this study, we showed for the first time that the H_2_O_2_-induced oxidative stress in MAC-T cells, a bovine MEC line, was accompanied with the disrupted redox homeostasis, endoplasmic reticulum stress, and cell apoptosis. We firstly provided evidence of potential utilization of resveratrol, a plant-derived polyphenolic compound, for ruminant medicine. We found that resveratrol exerts potent antioxidative potential through the induction of multiple antioxidant response genes. We further demonstrated that the beneficial effects of resveratrol appear to involve the upregulation of Nrf2 and activation of PI3K/Akt and ERK/MAPK signaling pathways (Figure 6).

Oxidative stress is widely recognized as the imbalance state between prooxidant and antioxidants. Both excessive production of ROS and deficiency of antioxidants resulted in endogenous oxidative stress [31]. Dairy cows undergo rapid metabolic and physiological adaptations during calving and early lactation, hallmarked by reduced levels of blood ascorbates and increased lipids [32]. Now it is becoming clear that dairy cows, especially in their mammary gland, suffer an oxidative state during this period [33], but the mechanisms by which bovine epithelial cells are damaged by oxidative stress have not been well studied [34, 35]. MAC-T cells have been extensively used as a bovine MEC model to study milk synthesis, lipid metabolism, and immune responses. In the present study, we firstly established an* in vitro* oxidative stress model in MAC-T cells by challenge H_2_O_2_. We found that incubation of MAC-T cells with 0.5 mM H_2_O_2_ for 24 h significantly damaged the cells with altered cell morphologic appearance, decreased cell viability, and increased ROS. This is consistent with several previous studies on BME-UV1, another bovine MEC line [34], and on primary bovine MECs [35]. Interestingly, we noticed that expressions of the unfolded protein response (UPR) regulators, GRP78 and CHOP, was strongly upregulated by the treatment with H_2_O_2_ for 4 hours, indicating that ER stress occurred in MAC-T cells. In addition, two major cell apoptosis-associated regulatory proteins, Bcl-2 and Bax, were also significantly affected in MAC-T cells after 24 h H_2_O_2_ treatment. Expression of Bcl-2, an apoptotic suppressor, was markedly decreased, whereas the expression of proapoptotic Bax gene was markedly increased, suggesting that H_2_O_2_ treatment increases cell apoptosis, which is consistent with the decreased cell viability in these cells. Our observation is supported by numerous studies documenting that persistent oxidative stress and ROS generation can elicit the protein misfolding and initiate apoptotic cascades, which are now known as important pathogenesis of multiple human diseases [36].

Because the loss of overall antioxidant potential has been linked to the decreased health and immune function in dairy cattle, especially during the transition period [37, 38], supplementing cattle feed with exogenous antioxidants to protect cattle against oxidative stress is under active investigation. For example, previous studies showed that supplementation of vitamin E [15] and Se [39] has a positive role in maintaining bovine immune functions [26]. Our previous studies also demonstrated that supplementation of antioxidants in early lactating cows can alleviate negative energy balance [40]. In addition, Liu et al. [41] recently found that caffeic acid, a natural polyphenolic antioxidant, showed a great* in vitro* anti-inflammatory effect in primary bovine MECs challenged by lipopolysaccharide. In the present study, we explored the effects of another well-known antioxidant, resveratrol [29, 42, 43], on the protection of MAC-T cells from oxidative stress.

First, we showed dose-dependent cytoprotective effects of resveratrol against H_2_O_2_-induced cytotoxicity and on the suppression of ROS accumulation in MAC-T cells. We found that H_2_O_2_-induced expression of GRP78 and CHOP was significantly reduced by resveratrol pretreatment, suggesting that resveratrol may restore the damaged ER homoeostasis by oxidative stress. In addition, resveratrol showed an antiapoptotic effect against oxidative stress by downregulating the Bax expression. It should be pointed out that resveratrol has versatile roles in several cell lines with respect to cell apoptosis and ER stress. In several human carcinogenic cell lines, such as colonic carcinoma cell lines HT29 [44] and COLO 201 [45], gastric adenocarcinoma cell line (SGC7901) [46], and hepatocellular carcinoma cell line (Hepa1-6) [47], resveratrol causes ER stress with upregulation of GRP78 and CHOP and a proapoptosis effect with elevated levels of phosphorylated elF2*α* and XBP-1 splicing. However resveratrol protects noncarcinogenic cardiac cell lines and diabetic patients against acute or chronic oxidative stress [28, 48]. These contradictory effects by resveratrol may be related to different ROS-mediated signaling and the intricate complexities between different networks of cell death, ER stress, and oxidative stress stimuli in different cells [36].

The antioxidant potential of resveratrol can be related to its free radicals scavenging activities by increasing the levels and activities of certain cellular noncatalytic antioxidant proteins. Thus, we examined gene expression of several major antioxidant/detoxificant enzymes involved in cytoprotection against oxidative stress. HO-1 and NQO-1 are two major principal phase II enzymes in promoting antioxidant activities [49, 50]. Cytosolic Txnrd is an important bovine antioxidant selenoproteins [51], which can reduce oxidized cysteine groups on proteins and functions in reducing both H_2_O_2_ and fatty acid hydroperoxides to less reactive water and alcohols [37]. In addition, efficient cell defense on the redox state is regulated by cystine/cysteine cycling and the cystine transporter, xCT [52]. Several previous studies have shown the inductive effects of resveratrol on these cellular redox-regulated proteins under physiological and pathological conditions [53, 54]. Thus, we analyzed the effects of resveratrol on the expression of these genes in MAC-T cells. We showed sequential transcriptional induction of these genes by resveratrol, starting with HO-1 at 4 h, followed by xCT, Txnrd-1, and NQO1. These results showed that resveratrol may restore MAC-T cells cellular redox state through the induction of these antioxidant genes (Figure 6).

Nrf2 is a transcription factor that plays a central role in the regulation of expression of several antioxidant/detoxifying enzymes through its interaction with ARE and protects the cells against cytotoxicity caused by oxidative stress. Nrf2 knockout mice are more sensitive to oxidative stress with decreased expression of ARE-regulated antioxidant genes [52]. Resveratrol is a well-known Nrf2 activator in several cellular models by disrupting the Nrf2/Keap1 interaction [55, 56]. In the present study, we hypothesized and tested whether resveratrol protects the bovine MECs against oxidative stress through Nrf2 activation. As expected, we found that resveratrol treatment increased both mRNA expression of Nrf2 and the nuclear accumulation of Nrf2 proteins in MAC-T cells. Furthermore, knockdown of Nrf2 abolished the induction of HO-1, Txnrd-1, and xCT by resveratrol under H_2_O_2_ treatment. These observations indicated that Nrf2 mediated the induction of HO-1, Txnrd-1, and xCT expression by resveratrol. It is worthy to note that Nrf2 knockdown in MAC-T cells resulted in more elevated expressions of GPR78 and CHOP under oxidative stress condition, implying a possible link between Nrf2 activation and ER stress.

In this study, we also found that resveratrol can activate ERK1/2 and Akt which are major signaling molecules involved in cell survival against oxidative stress [30, 48]. However, we did not observe significant changes in the phosphorylation of p38 and JNK by resveratrol treatment. In addition, resveratrol-induced gene expression of Nrf2, HO-1, and TrxR-1 was also inhibited by the specific inhibitors to ERK1/2 and Akt, but not by the inhibitors to p38 and JNK. These data suggested that ERK1/2 and Akt pathways also play an important role in mediating the protective effect of resveratrol. However, the inhibitor of p38 enhanced the expression of Nrf2 and several antioxidant genes, implying that p38 MAPK signaling may negatively regulate Nrf2 and cellular antioxidant responses, consistent with several previous studies [57]. p38 MAPK consists of four isoforms: p38*α*, p38*β*, p38*γ*, and p38*δ*. Activations of all of these isoforms have been reported to be associated with the dual phosphorylation of both threonine (Thr) and tyrosine (Tyr) residues of the target proteins [58]. The present study did not study which isoforms of p38 participated in the negative regulation of Nrf2 in MAC-T cells.

In summary, this study provided first-hand evidence of potential utilization of resveratrol in protection of oxidative stress in bovine MECs and investigated the underlying mechanisms. Treatment of resveratrol protected MAC-T cells against H_2_O_2_-induced oxidative stress by scavenging ROS formation and stimulating the Nrf2-ARE self-defense mechanism. In addition, resveratrol protected MAC-T cells against H_2_O_2_-induced cell apoptosis and ER stress. The antioxidant functions of resveratrol in MAC-T cells may be partially mediated by its activation of Akt and ERK signaling pathways. These beneficial effects of resveratrol provide us with its potential applications as a therapeutic ruminant medicine against oxidative insults.

## Supplementary Material

Supplemental Fig.1 Gene knockdown efficiency of candidate siRNAs in MAC-T cells .
Supplemental Fig 2. Effect of resveratrol on the cell viability of MAC-T cells.
Supplemental Fig. 3.Time effects of H2O2 treatment on the mRNA expression of GRP 78 and CHOP.
Supplemental Table 1: Sequences of primers used for quantitative real-time RT-PCR.
Supplemental Table 2. Sequences of siRNA duplex used for RNAi

## Abbreviations

ARE: Antioxidant response element
CHOP: C/EBP homologous protein
DCFH-DA: 2′,7′-Dichlorofluorescin diacetate
ER: Endoplasmic reticulum
GCLC: Glutamate cysteine ligase catalytic subunit
GRP78: Glucose-regulated protein 78
GSR: Glutathione reductase
HO-1: Hemeoxygenase 1
H_2_O_2_: Hydrogen peroxide
MECs: Mammary epithelial cells
Nrf2: Nuclear factor erythroid 2-related factor 2
NQO1: NADPH-Quinone oxidoreductase 1
PVDF: Polyvinylidene fluoride
ROS: Reactive oxygen species
SOD: Superoxide dismutase
Txnrd: Thioredoxin reductase
UPR: Unfolded protein response
xCT: Cysteine uptake transporter.
