# Supplementary material for: Protection of Bovine Mammary Epithelial Cells from Hydrogen Peroxide-Induced Oxidative Cell Damage by Resveratrol
Source: Oxid Med Cell Longev. 2015 Dec 28;2016:2572175. doi: 10.1155/2016/2572175 (PMC4707352; doi:10.1155/2016/2572175)
Supplement: Supplementary file 1 — Supplemental Fig.1 Gene knockdown efficiency of candidate siRNAs in MAC-T cells . Supplemental Fig 2. Effect of resveratrol on the cell viability of MAC-T cells. Supplemental Fig. 3.Time effects of H2O2 treatment on the mRNA expression of GRP 78 and CHOP. Supplemental Table 1: Sequences of primers used for quantitative real-time RT-PCR. Supplemental Table 2. Sequences of siRNA duplex used for RNAi [file 2572175.f1.docx]

# Supplementary Materials

Supplemental Fig.1 Gene knockdown efficiency of candidate siRNAs in MAC-T cells

MAC-T cells were transfected with 50 nM negative control siRNA-FAM(NC-FAM), negative control siRNA (NC) and Nrf2 candidate siRNAs for 24h, respectively. Cell transfection efficiency was confirmed by green fluoresce, from the cells transfected with NC-FAM (up panel). The knockdown efficiency of candidate Nrf2 siRNAs in MAC-T cell was confirmed by Nrf2 mRNA expression analysis after 24h trasfection (down panel). Gene expressions were measured using qRT-PCR, as described in Section 2. Results are shown as mean ± SD from three independent experiments and each value expressed as a target gene expression ratios comparing with the NC group. * Significantly different from NC control cells.
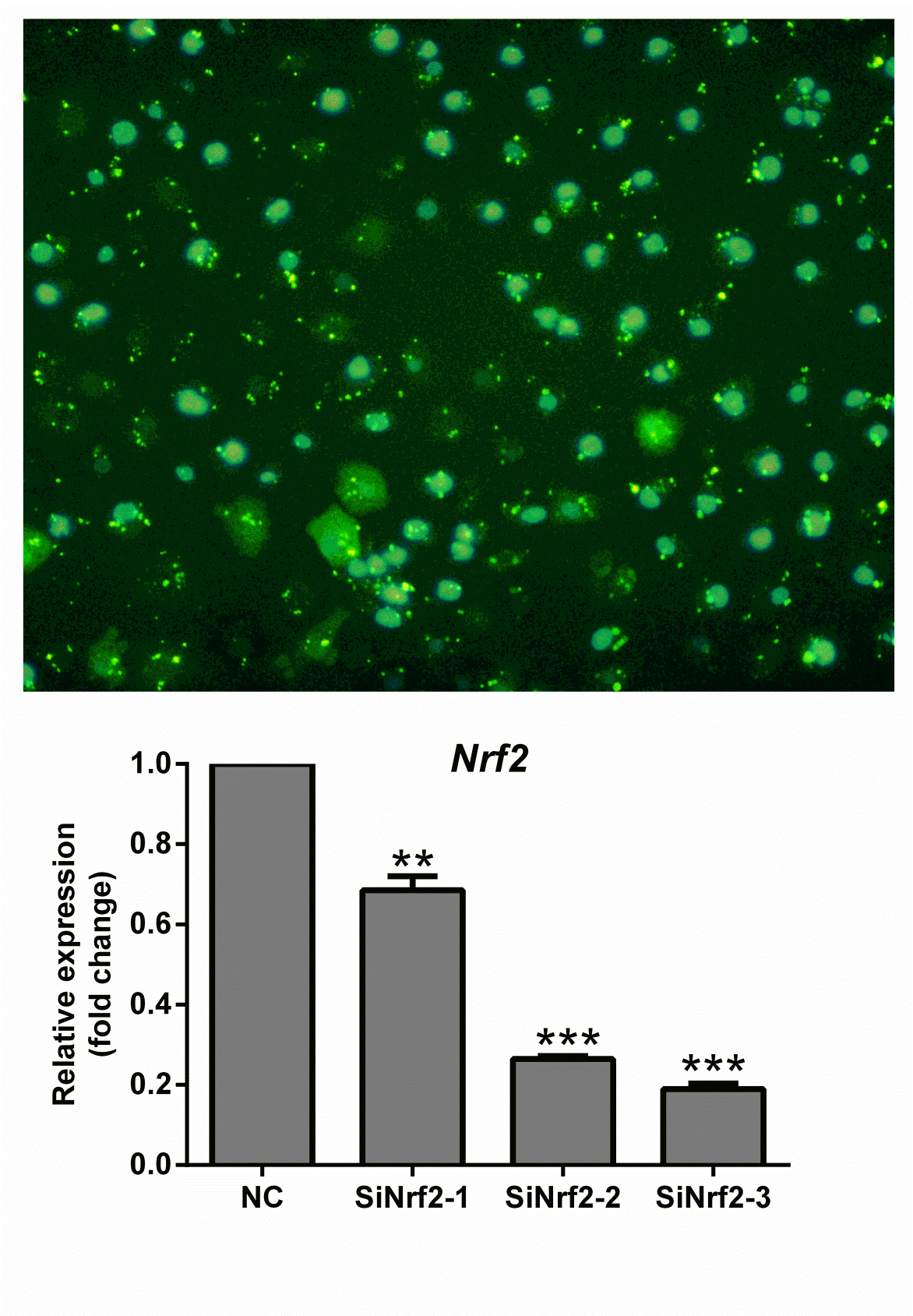


Supplemental Fig 2. Effect of resveratrol on the cell viability of MAC-T cells.

MAC-T cells were treated with increasing concentrations (0-100 μM) of resveratrol for 24 h and the viability of cells was determined by CCK-8 assay. Data are represented as mean ± SD of three experiments in which each treatment was carried out with at least 8 replicates. *p<0.05.


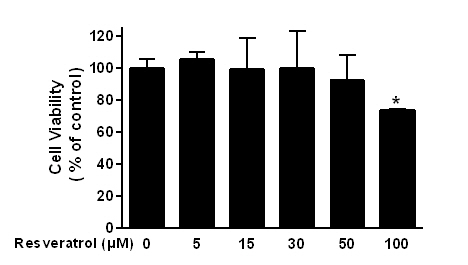


Supplemental Fig. 3.Time effects of H_2_O_2_ treatment on the mRNA expression of GRP 78 and CHOP

MAC-T cells were treated with 500 μM H_2_O_2_ for the indicated time periods. mRNA expressions of ER stress markers GRP 78 (A) and CHOP (B) were by real-time PCR. Data are represented as mean ± SD from three experiments.


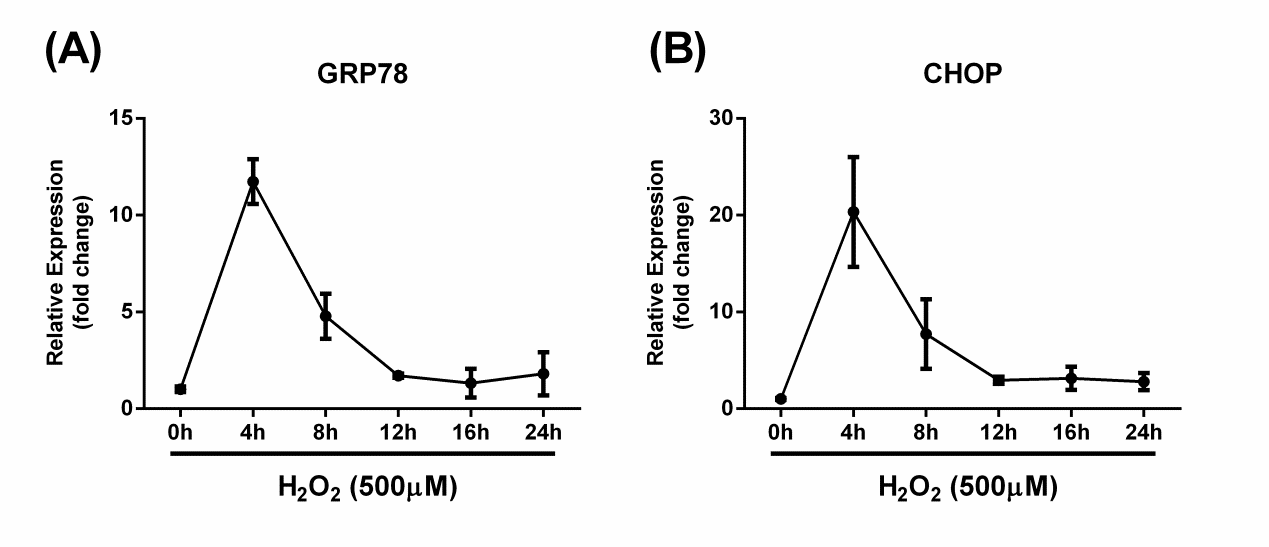


Supplemental Table 1: Sequences of primers used for quantitative real-time RT-PCR

| Gene | Primer sequence* | | Product size (bp) | GenBank Accession No. |
| --- | --- | --- | --- | --- |
| *TrxR-1* | F | 5’- GTGTTCACGACTCTGTCGGT -3’ | 240 | NM_174625.3 |
|  | R | 5’- CTGCCTTCCACGAATCACCT -3’ |  |  |
| *xCT* | F | 5’- GATACAAACGCCCAGATATGC -3’ | 136 | XM_002694373.2 |
|  | R | 5’- ATGATGAAGCCAATCCCTGTA -3’ |  |  |
| *CHOP* | F | 5’- TGAACGACTCAAACAGGAAATC -3’ | 248 | NM_001078163.1 |
|  | R | 5’- ACGCTAAGACCCTTTTCTATCG -3’ |  |  |
| *GRP78* | F | 5’- GACCCTGACTCGGGCTAAAT-3’ | 243 | NM_001075148.1 |
|  | R | 5’- TGGACAGCGGCACCATATG-3’ |  |  |
| *Bax* | F | 5’- TGGACATTGGACTTCCTTCG -3’ | 122 | NM_173894.1 |
|  | R | 5’- CCAGCCACAAAGATGGTCAC-3’ |  |  |
| *Bcl2* | F | 5’- GGATGACCGAGTACCTGAACC -3’ | 185 | NM_001166486.1 |
|  | R | 5’- GCCCAGATAGGCACCCAG -3’ |  |  |
| *β-actin* | F | 5’- CAAGGACCTCTACGCCAAC-3’ | 257 | NM_173979.3 |
|  | R | 5’- AGAAGCATTTGCGGTGGAC-3’ |  |  |
| *HO-1* | F | 5’- GGCAGCAAGGTGCAAGA-3’ | 221 | NM_001014912.1 |
|  | R | 5’- GAAGGAAGCCAGCCAAGAG-3’ |  |  |
| *NQO-1* | F | 5’- GGTGCTCATAGGGGAGTTCG -3’ | 235 | NM_001034535.1 |
|  | R | 5’- GGGAGTGTGCCCAATGCTAT -3’ |  |  |
| *Nrf2* | F | 5’- AGGACATGGATTTGATTGAC -3’ | 272 | NM_001011678.2 |
|  | R | 5’-TACCTGGGAGTAGTTGGCA -3’ |  |  |

*F=forward, R=reverse

Supplemental Table 2. Sequences of siRNA duplex used for RNAi

| **SiRNA ID** | **Target Position** | **Sequence*** | | | |
| --- | --- | --- | --- | --- | --- |
| siNrf2-1 | 620-642 | S 5': |  | GAGACUAGUACAGUUCCAA | dTdT |
|  |  | mRNA: | CT | GAGACTAGTACAGTTCCAA | GT |
|  |  | AS 3': | dTdT | CUCUGAUCAUGUCAAGGUU |  |
| siNrf2-2 | 492-514 | S 5': |  | CUGAAACUCUGAUCGUUCA | dTdT |
|  |  | mRNA: | AC | CTGAAACTCTGATCGTTCA | GG |
|  |  | AS 3': | dTdT | GACUUUGAGACUAGCAAGU |  |
| siNrf2-3 | 1434-1456 | S 5': |  | CAGUUGAGGACUUCAAUGA | dTdT |
|  |  | mRNA: | CC | CAGTTGAGGACTTCAATGA | AA |
|  |  | AS 3': | dTdT | GUCAACUCCUGAAGUUACU |  |
| NC/NC-FAM | / | S 5': |  | UUCUCCGAACGUGUCACGU | dTdT |
|  |  | mRNA: | / | / | / |
|  |  | AS 3': | dTdT | AAGAGGCUUGCACAGUGCA |  |

* S=sense, AS=anti-sense
